# Economic impact of implementing prescription of single-inhaler triple therapies *versus* current multiple-inhaler triple therapies for COPD in the Apulia Region

**DOI:** 10.1186/s12913-022-08640-9

**Published:** 2022-10-25

**Authors:** Emanuela Resta, Giulia Scioscia, Donato Lacedonia, Carla Maria Irene Quarato, Francesco Panza, Onofrio Resta, Giorgia Lepore, Enrico Buonamico, Valentina Di Lecce, Giovanna Elisiana Carpagnano, Maria Pia Foschino Barbaro, Noemi Rossi

**Affiliations:** 1 Translational Medicine and Health System Management, Department of Economy, University of Foggia, Foggia, Italy; 2 Department of Medical and Surgical Sciences, Institute of Respiratory Diseases, Policlinico Universitario “Riuniti” di Foggia, University of Foggia, Foggia, Italy; 3 Population Health Unit, “Salus in Apulia Study”, Research Hospital, National Institute of Gastroenterology “Saverio de Bellis”, Castellana Grotte, Bari, Italy; 4 Department of Basic Medical Sciences, Neuroscience and Sense Organs, Section of Respiratory Disease, University “Aldo Moro” of Bari, Bari, Italy; 5 Department of Law, Economics and Human Sciences (DIGIES) “Mediterranea”, University of Reggio Calabria, Reggio Calabria, Italy

**Keywords:** Economic impact, National Health Systems, COPD, Single-inhaler triple therapy, Multiple-inhaler triple therapy

## Abstract

**Background:**

The most impacting direct costs associated to COPD for the National Health Systems (NHS) are those related to accesses to the emergency room and hospital admissions, due to the onset of one or more COPD exacerbations. At the same time, severe COPD treatment, that often require a combination of medicaments, represents a substantial economic burden for the National Health Systems (NHS). This study aimed to evaluate the potential saving deriving from the implementation in the prescription of the two currently available single-inhaler triple therapies (SITTs) *versus* the currently used multiple-inhaler triple therapies (MITTs) in an eligible COPD population residing in the Apulia Region.

**Methods:**

A budget impact model was developed hypothesizing the progressive replacement of the different MITTs on the reference market (Scenario A) with the pre-established SITTs, assuming a degree of penetration of 30%, 50% and 100% (Scenario B). Drug costs were based on prices published on the Official Gazette and therapy durations were based on prescribing information over the year 2019 (IQVIA™ prescription dataset).

**Results:**

Our analysis showed that the extemporaneous MITT with the highest prevalence on the reference market was the inhaled corticosteroids/long-acting β_2_-agonists (ICS/LABA) combination plus a long-acting muscarinic antagonists (LAMA). This association of medicaments was paradoxically also the one associated to the highest expense value. The expanded use of a pre-established ICS/LAMA/LABA SITT was associated to a significant economic saving, ranging from a minimum of -€ 1,108,814 (SITT use: 30%) to a maximum of -€ 3,658,950 (SITT use: 100%). The cheapest pre-established SITT contained the fluticasone furoate/umeclidinium/vilanterol (FF/UMEC/VI) combination.

**Conclusion:**

A pre-fixed ICS/LAMA/LABA SITT is cost-saving, compared to the different currently used extemporaneous MITTs. Clinicians should consider the potential benefits of finding less expensive regimens while maintaining adequate efficacy in the prescriptive decision making process of COPD patients.

## Introduction

In coming decades, Chronic Obstructive Pulmonary Disease (COPD) is expected to globally increase because of the continued exposure to COPD risk factors and aging of the population. According to WHO estimates, COPD is projected to become the third leading cause of death worldwide for all age group in 2030 [1].

The most significant risk factor for COPD is long-term cigarette smoking, but also other environmental exposures such as biomass fuel exposure and air pollution may play a potential role. The natural history of the disease is characterized by exacerbations, progressive decline in pulmonary function and further concomitant chronic diseases such as cardiovascular diseases, osteoporosis, skeletal muscle dysfunction, metabolic syndrome, depression/anxiety and lung cancer. These aspects contribute to the overall disease severity, reduce health-related quality of life and make COPD one of the chronic progressive diseases with the highest socio-economic cost.

The actual annual cost of COPD in Italy was assessed for the first time by a Real Word Data (RWD) observational study conducted in a large region of northeast Italy (Triveneto), between 1999 and 2000 [2]. This study calculated an average annual per-patient treatment cost ranging from a minimum of € 1,500 to a maximum of € 3,913, depending on the severity of the disease. In 2008, the Social Impact of Respiratory Integrated Outcomes (SIRIO) study estimated an average annual per-patient treatment cost of € 2,723, with a range of values ​​that fluctuated from € 913 to € 5,452 [3]. More or less overlapping data were collected in another national multicenter study in 2007 [4]. In particular, the most impacting cost categories have been associated to hospital admissions and accesses to the emergency room, due to the onset of one or more COPD exacerbations. A patient with COPD and a history of previous exacerbations is estimated to impose a substantial burden on healthcare systems compared to a patient with no history of exacerbations. Patients with severe exacerbations have been shown to imply a higher cost than patients with a history of moderate exacerbations (by 27.8% and 48.1%, respectively) [5].

The goals of COPD pharmacologic therapy are reduction of symptoms and prevention of future exacerbations. Pharmacologic maintenance treatments can be categorized as inhaled corticosteroids (ICS), long-acting muscarinic antagonists (LAMA) and long-acting β_2_-agonists (LABA). According to the 2021 Global Initiative for Chronic Obstructive Lung Disease (GOLD) report, therapeutic regimens in which such treatments are often administered as combinations has to be adapted on the basis of the needs of each individual patient [6]. In particular, in COPD patients with a high level of symptoms despite a LABA/LAMA combination or who have persistent exacerbations despite an ICS/LABA association is recommended a triple therapy with an ICS, a LAMA and a LABA [6, 7].

One of the direct costs associated to COPD for the National Health Systems (NHS) is that related to treatment burden, that often require a combination of medicaments. Vice versa, frequent exacerbations, especially in severe COPD patients that are not optimally treated, are known to accelerate disease progression and mortality, frequently requiring several accesses to the emergency room and hospital admissions that further increase resource burden and healthcare costs [8].

Nowadays, two pre-established single-inhaler triple therapies (SITTs) are available: the fluticasone furoate/umeclidinium/vilanterol (FF/UMEC/VI) combination and the beclomethasone dipropionate/formoterol furoate/glycopyrronium (BDP/FOR/GB) combination. Both are indicated for maintenance treatment of moderate-to-severe COPD in adults who are not adequately treated with a combination of an ICS and a LABA or of a LABA and a LAMA.

On this background, the objective of our study was to estimate, from the Italian NHS perspective, the economic impact of the introduction in the market of these new SITT regimens *versus* a triple therapy administered *via* multiple inhalers (multiple-inhaler triple therapy [MITT]) in a population of COPD patients residing in the Apulia Region (Italy), aged ≥ 45 years and eligible for a triple therapy (being already under treatment with different extemporaneous MITTs).

## Methodology

### Model overview

A budget impact model (BIM) was developed to predict and understand the potential financial impact of implementing the use of the two nowadays available single-inhaler triple therapies (SITTs) in the current market share of the Apulia Region for the treatment of adult patients with moderate to severe COPD that require a maintenance treatment with an ICS plus LABA plus LAMA combination. The analysis estimated the costs of drugs from the perspective of the Italian Healthcare System.

The model compared the current treatment pattern in the Apulia Region with an alternative treatment pattern, in which an arbitrary growing proportion of suitable patients (equal to 30%, 50% and 100%, respectively) was assumed to switch from a MITT to a SITT over a study period ranging from January 1, 2019 to April 30, 2020. As a result, two scenarios are outlined. The first scenario (Scenario A) reflected a situation in which all the patients treated with a triple inhalation therapy were on either the different extemporaneous multiple-inhaler triple therapies (MITTs) or the two SITTs currently on the market, whereas the second scenario (Scenario B) assumed the progressive implementation of the two pre-established ICS/LAMA/LABA associations on the reference market in the target population.

In turn, Scenario B can be divided into three scenarios:

1. Scenario 1, in which was assumed a market penetration share of 30% for the pre-established SITTs *versus* a market penetration share of 70% for the extemporaneous MITTs.
2. Scenario 2, in which was assumed a market penetration share of 50% for the pre-established SITTs *versus* a market penetration share of 50% for the extemporaneous MITTs.
3. Scenario 3, in which was assumed a market penetration share of 100% for the pre-established SITTs *versus* a market penetration share of 0% for the extemporaneous MITTs.

This replacement process allowed to evaluate the reduction of the pharmaceutical expenditure that could be generated at a regional level by replacing the triple extemporaneous MITTs with the pre-established SITTs. The potential economic savings was quantified as the difference in total costs between the scenario of the current market shares (scenario A) and the hypothetical scenarios assuming the progressive implementation of the pre-established ICS/LAMA/LABA associations on the reference market (scenario B). The target population for the switch from a MITT to a SITT was defined as patients aged ≥ 45 years, residing in the Apulia region and already treated with an extemporaneous MITT.

Reporting of the study was guided by the established budget impact analysis-principles of good practice (ISPOR 2012) [9]. Given the purpose of the study and the therapeutic overlap of the pre-established SITTs compared to the open MITTs analyzed in the model (sharing the same “place in therapy”), no other accessory charges were assessed.

### Target population

For the definition of the cohort population in the study, all subjects aged ≥ 45 years and residing in the Apulia Region (as of January 1, 2017) were preliminarily identified from the National Statistical Institute (ISTAT) data. Subsequently, COPD patients were selected from the medical audit diagnosis service (reference period: 2017) and those who were under treatment with the different extemporaneous currently used MITTs were identified using the Longitudinal Prescription Database (LRx) of IQVIA™ for the Apulia Region regarding the pharmaceutical consumption of the different open triple therapies during the year 2019. The two datasets were carefully superimposed in order to avoid duplication or double counting of patients.

IQVIA™ Longitudinal Prescription Data (LRx) by IMS Health is a longitudinal patient prescription dataset based on retail pharmacy data. Thanks to a historical partnership with the National Federation of Italian Pharmacy Owners (FEDERFARMA) and the Federation of Italian Municipal Pharmacies (ASSOFARM), IMS Health gets monthly information on National Health System (NHS) reimbursed products (A classification). These data has to be communicated by all the territorial pharmacies to the Italian Ministry of Health in order to get reimbursement. IMS collects patient information reaching 80% of Retail channel and 60% of Direct Primary care (DPC) channel coverage. Collected information (updated as of April 30, 2020) included: encrypted date of birth, gender, age classes (5 years bound), product\pack dispensed and date of pharmacy dispensation. Patient information were leveraged in a statistical algorithm to infer a longitudinal anonymous patient ID.

Subjects with an inhalator triple therapy administered *via* similar or different devices were collectively referred as the “switch population” and were identified as the patients eligible for the switch to a treatment with the pre-fixed single-inhaler ICS/LAMA/LABA associations in the B scenario. Each patient was regarded as a patient on MITT at the recording moment of first association of the three single mono-components (an ICS a LABA and a LAMA) during the study period (i.e. over the year 2019). This moment represented the start date from which the patient was included in the budget impact analyzes.

### Treatment distribution

Users of the switch population were categorized according to the therapeutic regimen they were using.

The combination of drug therapies used by the switch population for Scenario A was defined on the basis of the market shares, calculated starting from IQVIA™ pharmaceutical consumption data and updated as of April 30, 2020. The reconstruction of the distribution was carried out on the basis of the average value per active ingredient/product.

For Scenario B, the number of patients eligible for the pre-established SITT was obtained by assuming a distribution of the market shares for the pre-established ICS/LAMA/LABA SITT *versus* the different extemporaneous MITTs currently used in proportion to a degree of penetration on the reference market equal to 30%, 50% and 100%, respectively.

### Treatment costs and adherence

The inhalation drugs of common use for the maintenance treatment of the COPD (comprising also the pre-established SITTs) are included in the lists of Class A medicinal products, which are reimbursed by the Italian NHS. The cost for the Italian NHS of each pharmacological therapy analyzed was therefore calculated by multiplying the cost of the individual package (obtained by applying the price to the public published in the Official Gazette and net of the compulsory discounts provided by law) for the duration of drug treatment (Table  1).

**Table 1:** Price to the public published in the Official Gazette and net of the compulsory discounts provided by law

COMBINATION	EX-FACTORY PRICE	PRICE TO THE PUBLIC
FF/UMEC/VI	€ 48.90*	€ 80.70*
BDP/FOR/GB	€ 55.54**	€ 91.67**

Abbreviations: FF: fluticasone furoate; UMEC: umeclidinium; VI: vilanterol; BDP: beclomethasone dipropionate; FOR: formoterol fumarate; GB: glycopyrronium bromide. Notes: Prices are to be intended net of the two cuts provided by Italian law. *GU n°58 of 09/03/2019; **GU n.1859 of December 20, 2019

Given the absence of data actually measured in an experimental setting or observed in the clinical reality, in our BIM we conservatively assumed that the adherence was equal to that of the other therapeutic alternatives analyzed (equity in adherence), i.e. equal to 6 packs/year per patient.

### Analysis

The total annual costs in both market scenarios (Scenario A and Scenario B) were estimated as the sum of product of the number of eligible patients treated with specific medication and the per-patient costs of specific medication over the duration of drug treatment. In the first scenario, the expense consisted of the cost determined by the treatment of patients with the different extemporaneous MITTs or the two pre-established SITTs currently on the market (Scenario A), while in the second hypothesis, the expense consisted in the cost of different portions of the target population considered treated with the pre-established ICS/LAMA/LABA associations and those of the remaining patients treated with the extemporaneous MITTs (Scenario B). The total budget impact for both the single inhaler combinations (FF/UMEC/VI and BDP/FOR/GB) was calculated as the difference in total costs between the two treatment scenarios (Scenario A - Scenario B), each of which represents the evolution of the other in relation to the pharmacological association under examination. An additional similar analysis was conducted to estimate the potential expenditure to be sustained in the market of the pre-established triple from the comparison between the FF/UMEC/VI and the BDP/FOR/GB associations.

## Results

### The “switch population”

**Fig. 1:**
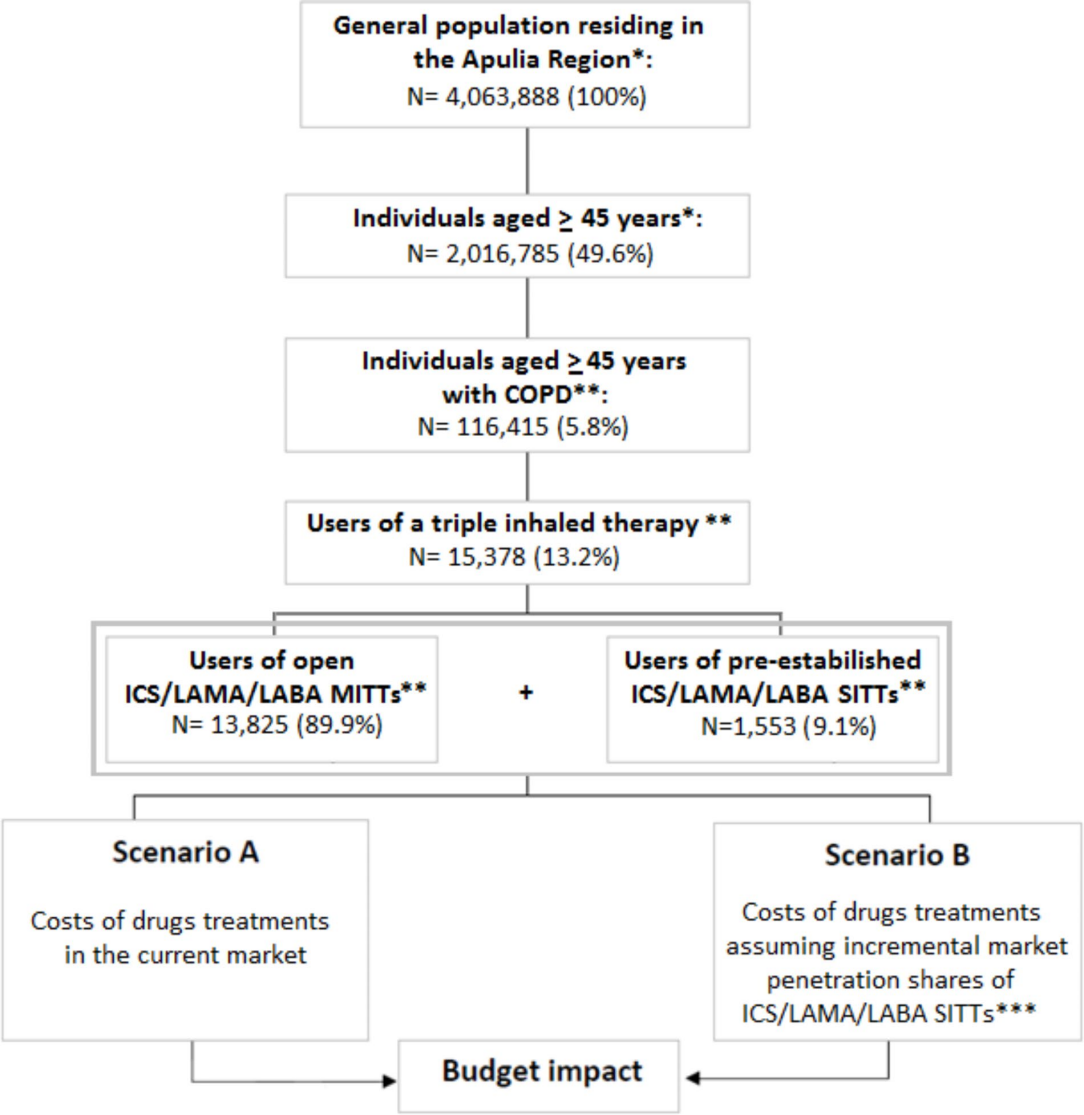
Identification of the “switch population”. Abbreviations: COPD: chronic obstructive pulmonary disease; MITT: multiple inhaler triple therapy; SITT: single inhaler triple therapy; ICS: inhaled corticosteroids; LABA: long-acting beta2-agonists; LAMA: long-acting antimuscarinic agents. *Data extraction from the National Statistical Institute (ISTAT) referring to the general and resident population in the Apulia Region surveyed on January 1, 2017; **Internal processing based on the prescriptive data offered by the Longitudinal Prescription Database (LRx) of IQVIA™ and combined with the medical audit diagnosis service (reference period: 2017); ***Costs were calculated assuming incremental market penetration shares (Market Share) of ICS/LAMA/LABA equal to 30%, 50% and 100%

Among a total of 4,063,888 subjects residing in the Apulia Region at 1 January 2017, 2,016,785 (49.6%) were aged ≥ 45 years. Of them, 116,415 (5.8%) had a diagnosis of COPD. A cohort of 15,378 (13,2%) COPD subjects was finally identified as being treated with an extemporaneous triple therapy taken through the use of one or more than one device. These subjects constituted the “switch population” eligible for our analysis (Fig. 1).

### Current reference market shares (a scenario)

**Fig. 2:**
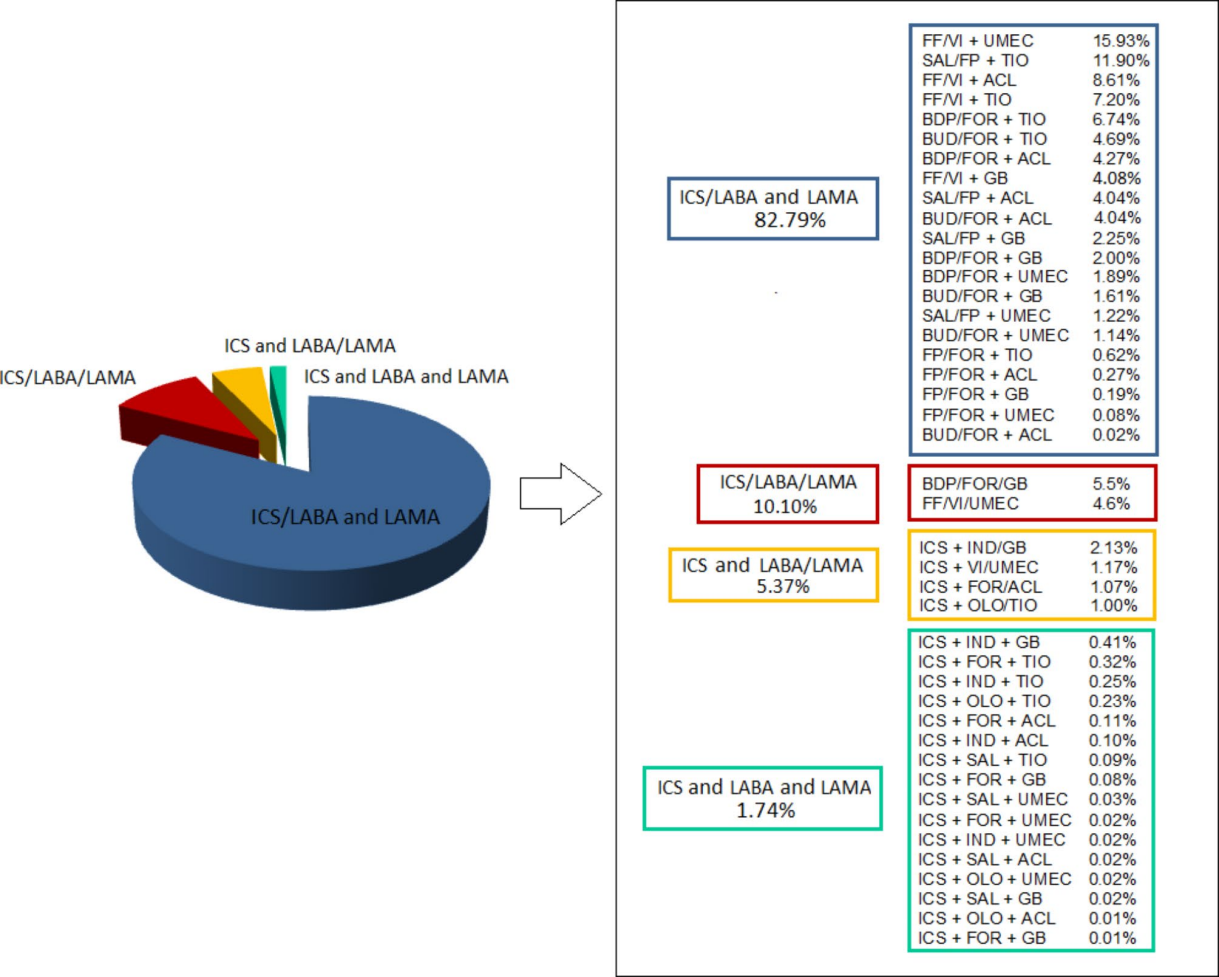
Market shares of the triple therapies currently used in the Apulia Region. Abbreviations: ICS: inhaled corticosteroids; LABA: long-acting beta2-agonists; LAMA: long-acting antimuscarinic agents; FF: fluticasone furoate; VI: vilanterol; UMEC: umeclidinium bromide; SAL: salmeterol; FP: fluticasone propionate; TIO: tiotropium bromide; ACL: aclidinium bromide; BDP: beclomethasone dipropionate; FOR: formoterol fumarate; BUD: budesonide; GB: glycopyrronium bromide; IND: indacaterol; OLO: olodaterol; “/”: drugs combination in a single inhaler; “+”: addition of a medication dispensed by another inhaler

Categorizing subjects of the switch population according to the therapeutic regimen they were using, clearly emerged that over 2/3 of the reference market was represented by the ICS/LABA combination plus the association of a LAMA (82.79%). More specifically, the therapeutic choices with the highest prevalence were represented by the following associations: Fluticasone Furoate/Vilanterol combination plus Umeclidinium Bromide (FF/VI + UMEC; 15.93%); Salmeterol/Fluticasone Propionate combination plus Tiotropium bromide (SAL/FP + TIO; 11.90%); Fluticasone Furoate/Vilanterol combination plus Aclidinium Bromide (FF/VI + ACL; 8.61%); Fluticasone Furoate/Vilanterol combination plus Tiotropium Bromide (FF/VI + TIO; 7.20%) and Beclometasone Dipropionate/Formoterol combination plus Tiotropium Bromide (BDP/FOR + TIO; 6.74%) (Fig.  2).

Among the extemporaneous MITTs, the therapeutic category with the lowest expense value was the triple association of an ICS a LAMA and a LABA dispensed *via* different inhalers, while the one with the highest expense value was the ICS/LABA combination plus a LAMA.

### Analysis in B scenario

**Fig. 3:**
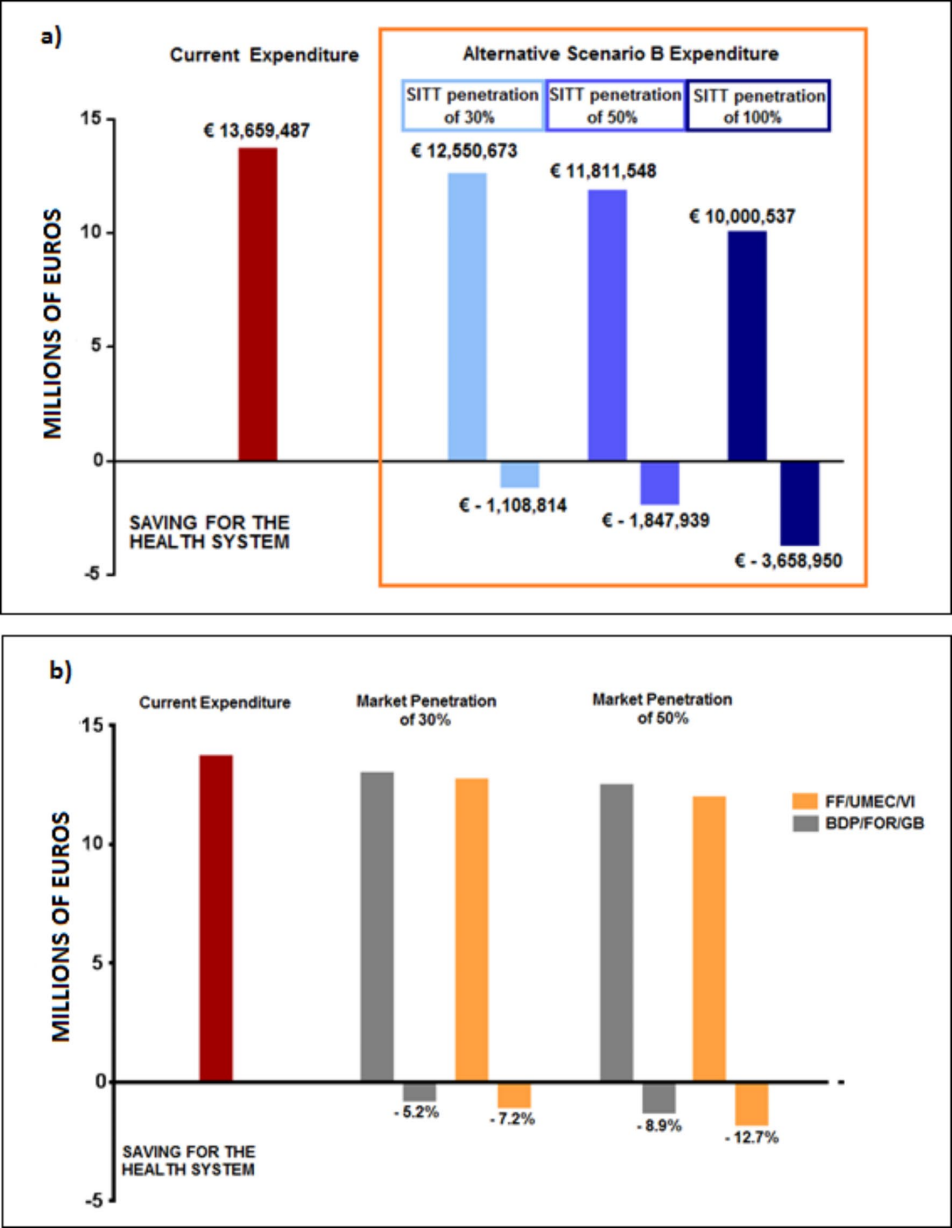
Analysis in scenario B. **a**) Pharmaceutical expenditure with related expected saving in proportion to the degree of penetration of the single-inhaler ICS/LAMA/LABA association on the market (with a penetration proportion of 30%, 50% and 100%, respectively). **b**) Reduction in expenditure for FF/UMEC/VI and for BDP/FOR/GB considering a penetration market share of 30%, and of 50%, respectively

In the hypothesized scenarios analyzed, the segmentation of the market in favor of the ICS/LAMA/LABA SITT, for both the FF/UMEC/VI and BDP/FOR/GB combinations, was inversely proportional to the degree of penetration and was associated with a significant reduction in pharmaceutical expenditure which ranged from a minimum of € 1,108,814 (Market Share ICS/LAMA/LABA: 30%) to a maximum of € 3,658,950 (Market Share ICS/LAMA/LABA: 100%). As for the potential economic advantages deriving from the commercialization of the pre-established SITTs compared to the other extemporaneous MITTs, an expected saving was observed directly proportional to the degree of penetration of the single-inhaler ICS/LAMA/LABA associations on the market (Fig.  3A).

Among the triple pre-established SITTs, the lowest expensive association was that containing FF/UMEC/VI. Indeed, compared to the scenario A (pharmaceutical expenditure incurred with the different extemporaneous MITTs currently used), there was a reduction in expenditure of 7.2% for FF/UMEC/VI and of 5.2% for BDP/FOR/GB considering a penetration market share of 30%, which reached up to 12.7% for FF/UMEC/VI and 8.9% for BDP/FOR/GB considering a market penetration of 50% (Fig.  3B).

## Discussion

COPD is a chronic and usually progressive lung disease. Although there is no cure for such disease, its progression may be slowed by smoking cessation and by avoiding exposure to other risk factors. In addition, an optimal treatment has been proven to reduce symptoms and exacerbation and enhance quality of life.

For COPD patients at high risk, with a persistent high level of symptoms and/or who experience continuous exacerbations despite a LABA/LAMA combination or an ICS/LABA treatment, GOLD guidelines recommends a triple therapy since theirs 2017 update [10]. However, when this recommendation appeared for the first time the possibility of a triple therapy administered through a single device was not yet available on the market. As a result, until recently, a triple therapy for COPD has been more frequently administered via multiple inhalers to be used at different times of the day.

Our analysis from IQVIA pharmaceutical consumption data have shown that the extemporaneous MITT with the highest prevalence on the market in the Apulia Region is currently represented by the ICS/LABA + LAMA combination (83.67%), that is also the one associated to the highest expense value. On the other side, our Budget Impact Model (BIM) has highlighted that the expanded use of a fixed ICS/LAMA/LABA association in a population of COPD patients residing in the Apulia Region, aged ≥ 45 years and eligible for a triple inhalation therapy contributes to an important reduction in the pharmaceutical expenditure, compared to the different currently used extemporaneous triple therapies. The aforementioned saving ranged from a minimum of - € 1,108,814 (Uptake ICS/LAMA/LABA: 30%) to a maximum of - € 3,658,950 (Uptake ICS/LAMA/LABA: 100%). The lowest expensive pre-established single-inhaler association was that containing FF/UMEC/VI, that has the advantage to require a single daily administration.

The main limitation of our analysis is that it relies only on the direct health costs associated with drug treatment. As with all chronic disease, the COPD associated economic burden may be related to both direct and indirect costs. Direct costs are those related to physician office visits, treatment, emergency room accesses, hospitalizations and rehabilitation. Indirect costs are commonly expressed as days off from work and refers to the morbidity and mortality caused by the diseases [11]. It is clear that the economic and social impact of COPD is greater than that of the only medications burden. However, the cost containment effect of a single-inhaler triple therapy would also be greater if other possible cost drivers were considered, such as the reduction in the rate of severe exacerbations and hospitalizations and the improvement of the patient’s adherence to therapy.

Three clinical trials of 1-year duration, the TRILOGY [12], TRINITY [13] and TRIBUTE [14] studies, have provided evidence for a clinical benefit of a pre-fixed twice daily administration extrafine triple therapy containing BDP/FOR/GB over LAMA monotherapy, ICS/LABA or LABA/LAMA treatment, with prevention of exacerbations being a key finding. Similarly, in the IMPACT study, the pre-fixed once daily administration triple therapy containing FF/UMEC/VI significantly reduced the rate of severe exacerbations compared with both dual therapies (LABA/LAMA and ICS/LABA) [15]. There is doubtless a strong association between severe exacerbations and hospitalization rate across all stages of COPD. Therefore, the entry on the market of the new single-inhaler triple therapies in the R03 class (drugs for obstructive respiratory tract syndromes) may entail further economic advantages associated, for example, with the reduction of the number of exacerbations which, as it is well known, are the cause of higher burdens associated with access to the emergency room and hospital admissions.

A recent Canadian study assessed the cost-effectiveness of a treatment with a single-inhaler triple therapy containing FF/UMEC/VI *versus* FF/VI or UMEC/VI by incorporating data from the IMPACT trial and using the GALAXY model to predict disease progression and outcomes [16]. In this study, despite the cost of such single-inhaler triple therapy was higher than that of both the dual comparator therapies, treatment with FF/UMEC/VI resulted to be more cost-effective than that with FF/VI or UMEC/VI. The result was explained by fewer severe exacerbations and associated hospital days, suggesting that the higher acquisition cost of FF/UMEC/VI could be partially offset by savings elsewhere in the health service. As a further confirm, other similar cost-effectiveness analyses conducted in the United Kingdom and in Spain and comparing FF/UMEC/VI with BUD/FOR reached the same conclusion [17], [18].

In this context, another aspect that deserves further study is represented by the evaluation of the additional economic benefit that may derive from an improved patients’ adherence to the treatment, that can now be delivered *via* a single device [19]. The therapeutic options currently include a single daily administration of fluticasone furoate, umeclidinium, and vilanterol (FF/UMEC/VI) delivered through the multi-dose dry powder Ellipta device [20] or a twice-daily dose of budesonide, glycopyrrolate and formoterol administered *via* a pressurised metered dose inhaler (pMDI) [20, 21]. Obviously, the choice of the device must take into account the preferences and characteristics of the patient [22–26].

Anyhow, patients’ adherence may also be linked to the treatment cost. To our knowledge, no study assessed the cost of common COPD medications and its impact on patients adherence. However, for a conditions like COPD in which adherence to expensive drugs is essential for preventing complications, it is important to find regimens that patients can afford without compromising efficacy. This highlights the importance of finding less expensive regimens while maintaining adequate efficacy. In this prospective, the strength of our study is to have showed that changing a patients who require a triple inhalation therapy to a regimen containing a pre-fixed ICS/LAMA/LABA combination in a single inhaler may imply drug cost savings to the healthcare system, as well as to the patient.

Another limitation of our study is that the analysis covered a period of only one year, while a budget impact model is usually calculated over a time horizon of 3 to 5 years. This limit is in line with the fact that our analysis was discontinued in conjunction with the emergence of the COVID-19 pandemic, also in consideration of the possible changes on the market resulting from the consequent health-care crisis. Anyhow, the COVID-19 pandemic has been shown to increase the therapy adherence and consequently the use of inhalers in patients with chronic respiratory diseases [27, 28]. On this basis, we could hypothesize that the proposed strategy of replacing the open MITTs with pre-established SITTs can cause also growing economic advantages over time. Comparison studies between the pre- and post-COVID 19 period would be interesting.

## Conclusion

In conclusion, our economic analysis found that a pre-fixed ICS/LAMA/LABA association in a single inhaler is cost-saving for the Italian NHS, compared to the different currently used extemporaneous triple therapies. Considering that the economic and social impact of COPD is greater than that of the only medications burden, this analysis appears to be only partial. However, several clinical trials evaluating the clinical outcomes of a triple therapy seems to be encouraging suggesting that the cost containment effect would also be greater if other possible cost drivers were considered. In this context, the entry on the market of the new single-inhaler triple therapies could favor the reduction in the number of COPD exacerbations, that have been clearly associated to the hospitalization rate. Additionally, the availability of single inhaler triple therapies may improve patients’ adherence to the treatment due to a better handling compared to the use of different devices and a favorable reduction in the cost of the medicine also for the patient. Clinicians should keep in mind these considerations in the prescriptive decision making process.

## Data Availability

The data that support the findings of this study are available from The Longitudinal Prescription Database (LRx) of IQVIA™ for the Apulia Region regarding the pharmaceutical consumption of the different open triple therapies during the year 2019 but restrictions apply to the availability of these data, which were used under license for the current study, and so are not publicly available. Data are however available upon payment from IMS Health (Official website: https://www.iqvia.com).

## Declarations

The study resulted from the analysis and the processing of datasets, where the data are properly anonymised. Due to the non-interventional nature of this study, Ethical approval was waived by the Ethics Committees of the Policlinico of Bari University Hospital and of the Policlinico “Riuniti” of Foggia University Hospital. All procedures were performed in accordance with relevant guidelines for a budget impact analysis (ISPOR 2012).

## Abbreviations

ACL: aclidinium bromide
BDP: beclomethasone dipropionate
BIM: Budget Impact Model
BUD: budesonide
COPD: Chronic Obstructive Pulmonary Disease
FF: fluticasone furoate
FOR: formoterol fumarate
FP: fluticasone propionate
GB: glycopyrronium bromide
ICS: inhaled corticosteroids
IND: indacaterol
LABA: long-acting beta2-agonists
LAMA: long-acting antimuscarinic agents
MITTs: multiple-inhaler triple therapies
NHS: National Health Systems
OLO: olodaterol
SAL: salmeterol
SITTs: single-inhaler triple therapies
TIO: tiotropium bromide
UMEC: umeclidinium bromide
VI: vilanterol
